# Supplementary material for: Dietary intake, nutritional status, and health outcomes among vegan, vegetarian, and omnivorous Czech families
Source: Commun Med (Lond). 2025 Nov 22;5:538. doi: 10.1038/s43856-025-01257-z (PMC12748659; doi:10.1038/s43856-025-01257-z)
Supplement: Supplementary file 2 — Supplementary Material [file 43856_2025_1257_MOESM2_ESM.pdf]

Supplementary Materials

Title: **Dietary intake, Nutritional status, and Health outcomes among Vegan, Vegetarian and Omnivorous Czech families**

Marina Heniková [1,2,*], Anna Ouřadová [1*], Eliška Selinger [1,3,4], Filip Tichanek [4], Petra Polakovičová [4], Dana Hrnčířová [2], Pavel Dlouhý [2], Martin Světnička [5], Eva El-Lababidi [5], Jana Potočková[1], Tilman Kühn [6], Monika Cahová [4†], Jan Gojda [1†]

1/ Department of Internal Medicine, Kralovske Vinohrady University Hospital and Third Faculty of Medicine, Charles University, Prague, Czech Republic;

2/ Department of Hygiene, Third Faculty of Medicine, Charles University, Prague, Czech Republic;

3/ Department of Epidemiology, Third Faculty of Medicine, Charles University, Prague, Czech Republic;

4/ Centre for Public Health Promotion, National Health Institute, Prague, Czech Republic ;

5/ Institute for Clinical and Experimental Medicine, Prague, Czech Republic;

6/ Department of Pediatrics, Kralovske Vinohrady University Hospital and Third Faculty of Medicine, Charles University, Prague, Czech Republic;

7/ Department of Epidemiology, MedUni, Vienna, Austria.

Correspondence:
Jan Gojda, MD, PhD., e-mail: jan.gojda@lf3.cuni.cz, ORCID no. 0000-0002-7995-5947

*These authors have contributed equally and shall be considered as joint first authors
† These authors jointly supervised this work

Supplementary results

Suppl. Table 1 *Medical history*

	VN	VG	OM
Adults (n=187)			
Allergy	12	12	17
Thyroid disease compensated	11	6	3
Type 1 DM on insulin	-	1	
Hypertension compensated	-	2	1
Hyperlipoproteinemia compensated	5	1	3
Osteoporosis	3	-	-
Children < 3 yo (n=77)			
Allergy	1	1	5
Atopy	-	1	4
Food intolerance	-	1	1
Iron deficiency anemia + neutropenia	-	-	1
Persistent foramen ovale	1	-	-
Children > 3 yo (n=65)			
Allergy	2	4	4
Atopy	1	1	2
Food intolerance	-	-	3
Gastroesophageal reflux disease	1	-	-
Persistent foramen ovale	1	-	-
Valve insufficiency	-	-	1
Umbilical hernia	-	-	1
Epilepsy	1	-	-
ADHD	1	-	-
Autism	1	-	-

Data are shown as number of subjects in each subgroup.

Suppl. Table 2 *History of fractures in adults.*

Description of the history of fractures per site in 91 vegan (VN), 45 vegetarian (VG), and 50 omnivore (OM) adult subjects. One subject from the VN group with osteogenesis imperfecta was excluded. N. s. = not specified.

	VN (n=91)	VG (n=45)	OM (n=50)
Subjects with any fracture (% portion)	n=36 (40%)	n=23 (51%)	n=27 (54%)
Total number of fracture events	n=53	n=39	n=37
Nose	1	1	3
Jaw	1	-	-
Cervical spine	1	-	-
Rib	3	-	-
Clavicle	2	3	6
Upper Arm	-	2	-
Forearm	7	2	6
Wrist	8	5	4
Metacarpals	2	1	1
Thumb/Fingers	7	7	7
Hand fracture (metacarpal/fingers) n. s.	5	-	1
Elbow	1	1	2
Kneecap	1	1	-
Tibia	1	2	3
Talus	-	1	-
Calcaneus	-	1	-
Ankle	5	2	1
Metatarsal	5	3	2
Big toe/Toes	2	1	-
Foot n. s.	1	-	1

Suppl. Table 3 *Demographics and physical activity in adults.*

n=164	VN	VG	OM
Area of residence by number of inhabitants			
>1 000 000	34	18	32
50 000-1 000 000	12	8	2
20 000-49 999	4	0	0
5000-19 999	14	4	2
1000-4999	12	6	4
< 999	8	2	2
Level of education			
Primary education or lower	2	1	0
Secundary edudation (including vocational education or vocational training)	28	10	8
Bacherlor or higher education	54	27	34
Physical activity			
Baecke score (total score)	7,9 (6,81;9,63)	7,79 (7,27;9,51)	8,09 (7,42;9,24)

Description of the area of residence, education and physical activity in vegan, vegetarian and omnivore adults. Data are shown as number of subjects. Baecke score (total score) as medians (25th; 75th percentile), https://doi.org/10.1590/S1517-86922003000300002

Suppl. Table 4 *Dietary intake in children <3 years old among dietary groups.*

		VN	VG	OM	Kruskal-Wallis test			Wilcox multiple comparison test					
					chi-squared	df	p_val	VN_OM		VG_OM		VN_VG	
								W stat	p_val	W stat	W stat	p_val	W stat
	Energy kcal . day^{-1}	1,008 (665; 1,349)	980 (810; 1,087)	856 (761; 1,017)	1.581	2	0.453						
macronutrients	Proteins g . day^{-1}	30 (16, 37)	29 (19, 34)	29 (21, 36)	0.275	2	0.872						
	Carbohydrates g . day^{-1}	122 (85, 168)	119 (88, 146)	104 (89, 114)	2.684	2	0.261						
	Sugars g . day^{-1}	60 (49, 75)	58 (46, 74)	51 (45, 63)	1.415	2	0.492						
	Fiber g . day^{-1}	15 (8, 19)	12 (5, 17)	8 (5, 12)	5.219	2	0.074						
	Fat g . day^{-1}	38 (30, 48)	37 (33, 46)	36 (23, 40)	1.805	2	0.406						
	Saturated fat g . day^{-1}	5.2 (3.2, 7.8)	5.2 (3.3, 8.4)	9.5 (6.5, 14.5)	6.864	2	0.032	355	0.008	114	0.109	185	0.816
	Cholesterol mg . day^{-1}	52 (0, 103)	91 (52, 117)	101 (52, 121)	2.779	2	0.057						
micronutrients	Selenium µg . day^{-1}	8.9 (7.7, 11.2)	8.4 (7.7, 18.3)	12.0 (10.0, 17.1)	5.746	2	0.2						
	Zinc mg . day^{-1}	4.36 (2.64, 5.84)	2.86 (2.45,4.49)	2.90 (2.34, 4.16)	3.471	2	0.3						
	Iron mg . day^{-1}	3.69 (1.81, 5.42)	2.64 (1.40,4.46)	2.33 (1.48, 3.64)	2.352	2	0.7						
	Phosphorus mg . day^{-1}	357 (209, 584)	313 (199, 520)	345 (308, 549)	0.745	2	0.5						
	Magnesium mg . day^{-1}	124 (76, 191)	90 (54, 146)	90 (58, 145)	0.507	2	0.6						
	Calcium mg . day^{-1}	337 (271, 401)	286 (271, 368)	337 (293, 540)	0.601	2	0.6						
	Iodine µg . day^{-1}	42 (29, 52)	50 (32, 53)	50 (27, 57)	1.031	2	0.9						

Medians (25th; 75th percentile) of dietary intake in vegan (VN), vegetarian (VG) and omnivorous (OM) adults. The overall difference among groups was evaluated using Kruskal-Wallis test (KW) followed by Mann-Whitney U test. All P-values are two-sided; no adjustment for multiple comparisons was applied.

Suppl. Table 5 *Dietary intake in children ≥3 years old among dietary groups.*

		VN	VG	OM	Kruskal-Wallis test			Wilcox multiple comparison test					
					chi-squared	df	p_val	VN_OM		VG_OM		VN_VG	
								W stat	p_val	W stat	W stat	p_val	W stat
	Energy kcal . day^{-1}	1,250 (1,112; 1,398)	1,369 (1,166; 1,555)	1,386 (1,132; 1,690)	0.968	2	0.6						
macronutrients	Proteins g . day^{-1}	42 (30, 45)	42 (36, 56)	50 (46, 60)	6.005	2	**0.049**	206	**0.011**	163	0.189	140	0.446
	Carbohydrates g . day^{-1}	172 (142, 195)	166 (131, 217)	158 (135, 179)	1.234	2	0.6						
	Sugars g . day^{-1}	54 (45, 66)	45 (40, 56)	71 (50, 89)	5.985	2	**0.049**	176	0.157	190	**0.018**	92	0.281
	Fiber g . day^{-1}	26 (18, 31)	19 (13, 23)	14 (14, 16)	11.370	2	**0.003**	49	**0.001**	73	**0.040**	78	0.101
	Fat g . day^{-1}	39 (37, 42)	47 (39, 52)	51 (36, 66)	1.650	2	0.4						
	Saturated fat g . day^{-1}	7 (5, 10)	14 (11, 21)	20 (16, 26)	20.200	2	**<0.001**	248	**<0.001**	173	0.089	204	**<0.001**
	Cholesterol mg . day^{-1}	0 (0, 0)	53 (13, 82)	111 (77, 163)	16.690	2	**<0.001**	249	**<0.001**	164	0.176	182	0.027
micronutrients	Selenium µg . day^{-1}	10 (8, 17)	14 (10, 26)	20 (16, 33)	7.543	2	**0.021**	207	**0.010**	174	0.082	151	0.232
	Zinc mg . day^{-1}	4.81 (3.00, 7.34)	3.53 (3.20, 4.97)	4.65 (3.83, 6.53)	2.031	2	0.4						
	Iron mg . day^{-1}	6.23 (5.01, 8.66)	5.22 (4.36, 6.36)	5.61 (5.12, 7.33)	2.019	2	0.4						
	Phosphorus mg . day^{-1}	541 (405, 769)	667 (469, 804)	637 (598, 892)	2.555	2	0.3						
	Magnesium mg . day^{-1}	239 (165, 320)	156 (140, 209)	163 (129, 185)	5.243	2	0.077						
	Calcium mg . day^{-1}	346 (327, 481)	476 (427, 588)	495 (309, 662)	2.593	2	0.2						
	Iodine µg . day^{-1}	21 (15, 38)	38 (23, 57)	38 (24, 54)	4.240	2	0.14						

Medians (25th; 75th percentile) of dietary intake in vegan (VN), vegetarian (VG) and omnivorous (OM) adults. The overall difference among groups was evaluated using Kruskal-Wallis test (KW) followed by Mann-Whitney U test. All P-values are two-sided; no adjustment for multiple comparisons was applied. p<0.05 are in bold.

Suppl. Table 6 *Dietary intake in adults among dietary groups.*

		VN	VG	OM	Kruskal-Wallis test			Wilcox multiple comparison test					
					chi-squared	df	p_val	VN_OM		VG_OM		VN_VG	
								W stat	p_val	W stat	p_val	W stat	p_val
	Energy kcal . day^{-1}	2,247 (1,846; 2,769)	2,246 (1,717; 2,642)	2,029 (1,722; 2,377)	4.074	2	0.130						
macronutrients	Proteins g . day^{-1}	75 (65; 97)	70 (60; 103)	84 (74; 106)	2.091	2	0.352						
	Carbohydrates g . day^{-1}	284 (228; 337)	221 (199; 285)	214 (182; 248)	21.190	2	**<0.001**	814	**<0.001**	563	0.354	891	**0.002**
	Sugars g . day^{-1}	65 (50; 80)	63 (44; 99)	62 (46; 86)	0.221	2	0.896						
	Fiber g . day^{-1}	38 (30; 52)	32 (23; 42)	20 (16; 26)	51.169	2	**<0.001**	303	**<0.001**	295	**<0.001**	984	**0.013**
	Fat g . day^{-1}	72 (60; 95)	77 (65; 97)	77 (60; 92)	0.174	2	>0.9						
	Saturated fat g . day^{-1}	14 (10; 19)	23 (14; 31)	30 (24; 37)	43.536	2	**<0.001**						
	Cholesterol mg . day^{-1}	0 (0; 1)	56 (8; 81)	164 (137; 231)	55.182	2	**<0.001**	2856	**<0.001**	1035	**<0.001**	1787	0.172
micronutrients	Selenium µg . day^{-1}	22 (15; 34)	23 (16; 37)	37 (25; 47)	17.635	2	**<0.001**	2294	**<0.001**	903	0.007	1483	0.591
	Zinc mg . day^{-1}	11.2 (6.7; 14.8)	8.0 (5.0; 11.6)	8.5 (6.6; 10.8)	7.667	2	**0.021**	1182	**0.034**	713	0.456	1012	**0.021**
	Iron mg . day^{-1}	13 (10; 20)	11 (7; 14)	9 (8; 12)	18.204	2	**<0.001**	856	**<0.001**	585	0.497	939	**0.012**
	Phosphorus mg . day^{-1}	1,087 (812; 1,564)	1,110 (792; 1,394)	1,186 (987, 1;397)	1.421	2	0.491						
	Magnesium mg . day^{-1}	437 (301; 605)	361 (255; 474)	299 (255; 339)	18.144	2	**<0.001**	823	**<0.001**	470	**0.047**	1072	0.051
	Calcium mg . day^{-1}	711 (555; 948)	773 (563; 1,057)	881 (684; 1,073)	5.798	2	0.055						
	Iodine µg . day^{-1}	59 (40; 82)	64 (47; 69)	73 (60; 86)	8.424	2	**0.015**	2045	**0.006**	849	**0.022**	1449	0.741

Medians (25th; 75th percentile) of dietary intake in vegan (VN), vegetarian (VG) and omnivorous (OM) adults. The overall difference among groups was evaluated using Kruskal-Wallis test (KW) followed by Mann-Whitney U test. All P-values are two-sided; no adjustment for multiple comparisons was applied. p<0.05 are in bold.

Suppl. Table 7 *Supplementation habits among dietary groups across age strata.*

Children < 3 years old							
Supplement	VN	VG	OM	p-value	OM vs VG	OM vs VN	VG vs VN
Vitamin B12	31 (77.5%)	7 (47%)	-	<0.001	0.001	<0.001	0.049
Vitamin D	34 (85%)	14 (93%)	12 (55%)	0.015	0.03	0.03	n.s.
Magnesium	-	-	-	N/A			
Zinc	1(2.5%)	-	-	n.s.			
Selenium	2(5%)	-	-	n.s.			
Calcium	1(2.5%)	-	-	n.s.			
Iron	2(5%)	1 (6.7%)	1 (4.5%)	n.s.			
Iodine	3(7.5%)	-	-	n.s.			
ω3-fatty acids	23(57.5%)	2 (13%)	2 (9.1%)	<0.001	n.s.	0.001	0.006
Children ≥ 3 years old							
Supplement	VN	VG	OM	p-value	OM vs VG	OM vs VN	VG vs VN
Vitamin B12	18(85.7%)	19(86.4%)	-	<0.001	<0.001	<0.001	n.s.
Vitamin D	17(81%)	20(90.9%)	9 (41%)	0.005	0.006	n.s.	n.s.
Magnesium	3(14.3%)	4 (18.2%)	-	n.s.			
Zinc	4(19%)	-	-	N/A			
Selen	4(19%)	4 (18.2%)	-	N/A			
Calcium	2(9.5%)	2 (9.1%)	-	n.s.			
Iron	2(9.5%)	3 (13.6%)	1 (4.5%)	n.s.			
Iodine	6(28.6%)	7(31.8%)	-	n.s.			
ω3-fatty acids	16(76.2%)	11 (50%)	3 (14%)	<0.001	0.016	<0.001	n.s.
Adults							
Supplement	VN	VG	OM	p-value	OM vs VG	OM vs VN	VG vs VN
Vitamin B12	79(85.9%)	27(60%)	1 (2.0%)	<0.001	<0.001	<0.001	0.001
Vitamin D	68(73.9%)	35(77.8%)	17 (34%)	<0.001	<0.001	<0.001	n.s.
Magnesium	12 (13%)	11 (24.4%)	13 (26%)	n.s.			
Zinc	5 (5.4%)	6 (13.3%)	6 (12%)	n.s.			
Selen	7(7.6%)	7(15.6%)	-	n.s.			
Calcium	10 (10.9%)	3 (6.7%)	-	n.s.			
Iron	12 (13%)	3 (6.7%)	3 (6.0%)	n.s.			
Iodine	15(16.3%)	8(17.8%)	1 (2.0%)	n.s.			
ω3-fatty acids	47 (51.1%)	14 (31.1%)	6 (12%)	<0.001	0.028	<0.001	0.028

Supplements' use in vegans (VN), vegetarians (VG), and omnivores (OM) across age strata. Data are presented as absolute numbers of self-reported regular supplementers in each group and their proportion compared to the total number of subjects enrolled in each subgroup. The differences among groups was evaluated using Kruskal-Wallis test (KW) followed by Mann-Whitney U test.

Supplementary Methods

Design and the study population

The current study is the output of a baseline cross-sectional study that was performed as the initiation of the prospective cohort study among families KOMPAS: Cohort prospective study of emerging nutritional factors among families. The details of the prospective study protocol can be found at (Selinger et al., 2024). Families consisting of two adults and at least one child under 7 years of age (if another older child was in the family, she/he was also enrolled) with the same dietary eating pattern (self-identified vegan, vegetarian, or omnivore) were recruited and enrolled. A vegan diet (VN) was defined as a diet excluding meat/meat products, fish, milk, dairy, and eggs. A vegetarian diet (VG) was defined by the exclusion of meat/meat products and fish, but consuming eggs or dairy on a daily basis. An omnivore diet (OM) was defined as a diet with no dietary restrictions put on food of animal origin, consuming meat products, fish, eggs, and dairy daily. Diet group membership of the whole family was determined by self-identification. The Exclusion criteria consisted of a different diet in individual family members, any disease associated with malabsorption (pancreatitis, celiac disease, phenylketonuria, etc), and inability or unwillingness to undergo full clinical examination and biospecimen sampling.

Data collection

Clinical visit, structured medical history, survey

During the clinical visit, physicians took a detailed structured history; in adults, focusing on dietary habits and records of events related to bone health (fractures), iodine status (iodine deficiency, goiter), cardiometabolic health (overweight, obesity, diabetes, cardiovascular events, dyslipidemia, hypertension), and thyroid disease (autoimmune thyroiditis); in children, focusing on dietary habits, birth and breastfeeding history, psychomotor development, autoimmune diseases (IBD, type 1 diabetes, atopy). Adult participants completed a brief survey focusing on basic sociodemographic data, lifestyle and dietary habits.

Anthropometrics

Height and weight were measured by trained health professionals for all family members, while muscle strength (maxHGS), blood pressure, body composition (BIA, Nutriguard-M, Data Input GmbH, Tanita MC-780 MA), and waist and hip circumference were measured only for adults. The height of infants and toddlers was measured with an infantometer, and the height of older children and adults was measured with a stadiometer. The measurements were then converted into percentiles using standard percentile charts validated for use in the Czech Republic (Vignerová & Lhotská, 2006).

Laboratory samples

Venous blood was obtained after an overnight fast (12 hours for adults and non-breast-fed children) from the antecubital vein was performed. Tubes were collected, centrifuged and aliquoted, kept refrigerated, and stored at −80°C during the same morning until being analyzed. Spot urine samples were also collected at the clinical visit, kept refrigerated, and stored at −80°C during the same morning until being analyzed. Laboratory parameters of interest included routine clinical markers, glucose and lipid metabolism parameters (glucose, cholesterol, C-LDL, C-HDL, triglycerides), iron metabolism parameters (blood count, iron, ferritin, total iron binding capacity: TIBC, transferrin, saturation of transferrin), calcium-phosphate and bone metabolism parameters (Calcium, Phosphorus, Parathormone: PTH, 1,25-hydroxy vitamin D, N-terminal pro-peptides of type 1 collagen: P1NP, beta cross-laps: CTx, ALP), vitamin B12

metabolism parameters (active B12: Holotranscobalamine, Homocysteine, Methylmalonic acid: MMA, Folate), and iodine metabolism parameters (Urine Iodine Concentration). All laboratory parameters were analysed in ISO-certified institutional laboratories. All analytic methods and analyzers are summarized in **Suppl. Data 1**.

Nutritional assessment

A 3-day weighted dietary record method was used to evaluate the dietary intake. Each participant received education on accurately recording dietary intake from a dietitian during a personal visit. Parents were responsible for weighing and documenting all foods and beverages consumed by their children and their own intake over three days, including two weekdays and one weekend day. Electronic kitchen scales were used. In instances where precise weighing was not feasible, such as when dining out, household measures and a photographic booklet displaying portion sizes were used for semi-quantitative recording. The dietitian reviewed any missing data and contacted participants electronically to gather the necessary information.
Breast milk intake was recorded by mothers in terms of breastfeeding duration in minutes. Based on this, an estimated intake of breast milk in milliliters was calculated, considering the child's age and weight. This calculation utilized information from a meta-analysis providing an estimate of the average daily amount of milk a child might consume at various ages (Rios-Leyvraz & Yao, 2023).
Nutrient and energy intake data were analyzed using the NUTRIXO nutritional software (ArcaiSoft, Czech Republic). This software comprises validated EuroFIR databases, and the national and other international food composition databases (FCDBs). For products used exclusively in Europe, such as infant formula or specific vegan products newly introduced to the Czech market and not listed in any of the validated databases, including the Czech database (Nutridatabaze.cz), the dietitian recorded nutrient content from the product packaging.

Statistical methods

All statistical analyses were conducted using R, version 4.4.0 (2024-04-24) (R Core Team 2023, https://www.R-project.org/). Data were visualized mainly with the help of *ggplot2* and *ggpubr* (Kassambara, 2023) R packages.

Summary statistics

Differences in numerical clinical outcomes were assessed using robust linear mixed-effects (rLME) models using ‘robustlmm’ package (Koller, 2016) separately for children and adults, adjusting for pre-specified key confounders, selected based on domain knowledge and literature, selecting these with their known association with clinical characteristics and observed differences in distribution among dietary groups. This approach was chosen to avoid data-driven selection, which can bias estimates by overfitting or introducing collider bias. Specifically, we included age (log2-transformed for children), sex, and breastfeeding-related covariates (exclusive breastfeeding [0/1], partial breastfeeding [0/1], and breastfeeding duration in months), along with a random intercept for family to account foe within-family dependency. Where relevant (e.g., for biogenic elements and vitamins), supplementation status was also included. For children’s morphological characteristics, birth weight was an additional covariate. If diagnostic checks indicated non-normality or heteroscedasticity of residuals, we used a log2 transformation of the outcome when helpful.

We likewise fitted conventional linear mixed-effects models (LME) via ‘lme4’ (Bates et al., 2015) to evaluate importance of all variables. Specifically, we compared models with and without each variable group (diet, sex, age, importance of breastfeeding-related variables in the case of children, random effect of family) using the Akaike Information Criterion (AIC), which estimates how well a model is expected to predict new data. A drop in AIC when a variable group was removed indicates that it contributes to improving the predictive performance of the model.

For (r)LME, observations with missing outcome data were excluded from analysis, assuming missingness was unrelated to diet group. Thirteen missing values for partial breastfeeding were imputed using a regression-based approach, finally assigning 0 if a child was <1.77 years and 1 otherwise, reflecting its strong relationship with age.

The modeling process included:

1. Fitting a random-intercept generalized additive model (GAMM) with age as a non-linear predictor using ‘mgcv’ (Wood, 2011)
2. Checking residuals and refitting with a log2-transformed outcome when needed.
3. Testing linear vs. quadratic age effects in subsequent (r)LME models if nonlinearity was indicated.
4. Applying robust LME modeling.
5. Conducting further mixed effects model with lme4 package, excluding specific covariates, or random effect of family, to evaluate their importance for each clinical outcome prediction. The Akaike Information Criterion (AIC) measured covariate importance regarding estimated out-of-sample predictive accuracy. A decrease in AIC after a covariate’s inclusion suggests an improvement in the model’s predictive capability, indicating importance of given variable.
6. In case when AIC is reduced with the inclusion of the random effect of family, we also calculated inter-class correlation, showing how much are observations correlated within family after controlling the effect of other variables, and thus strength of within-family clustering.

We visualized results using volcano plots (adjusted standardized differences between diet groups) and heatmaps (AIC changes after covariate removal). Significance level of $\alpha = 0.05$ ($P < 0.05$) was considered ‘significant’. Raw p-values (not corrected for multiple comparisons) are reported to maximize sensitivity for a potential risk associated with the vegan/vegetarian diet (omitting a true risk was considered more serious than allowing a few false positives). However, FDR-corrected P-values and confidence intervals for the diet group differences can be found in the online statistical report: https://filip-tichanek.github.io/kompas_clinical/.

For binary outcomes, we applied logistic generalized linear additive mixed-effects models (GAMM), reporting odds ratios (OR).

Diet prediction

To assess the predictive power of clinical outcomes on diet strategy, we employed Elastic Net logistic regression using the ‘glmnet’ R package (Friedman et al., 2010)

For both adults and children, we first fitted a ***baseline model*** incorporating basic subject characteristics (age, sex, and, for children, breastfeeding status) as predictors. We then expanded the analysis with a ***reduced model*** that included these basic characteristics along with diverse clinical outcomes not primarily affected by supplementation. Finally, we fitted a ***full model***, incorporating all clinical characteristics, including those strongly influenced by supplementation.

Missing predictor values were imputed using predictive mean matching (single stochastic imputation) with the ‘mice’ R package (van Buuren & Groothuis-Oudshoorn, 2011) All numerical predictors were standardized by dividing by 2 standard deviations using the ‘arm’ R package (Gelman & Su, 2024) to ensure scale comparability.

The predictive performance of the models was evaluated based on their ability to discriminate between diet groups in out-of-sample data, using the area under the ROC curve (AUC) as the measure of discriminatory capacity (estimated with the 'pROC' R package (Robin et al., 2011).To achieve this, we applied a cluster bootstrap resampling method (500 simulations), maintaining family-wise integrity in training and testing sets (i.e., all members of a single family were included in either the training or testing sample in each iteration) to prevent data leakage and overestimation of accuracy. This validation procedure was implemented using custom functions.

Estimated accuracies (AUC values) were compared against the baseline model to assess whether the more complex models provided a significant AUC gain. A model was considered to offer a significant improvement if the lower bound of the 95% confidence interval for the difference in AUC (complex model minus baseline model) was above zero.

The process of building the elastic net models and estimating accuracy involved the following steps:

1. The cv.glmnet function from the 'glmnet' package was utilized to determine the optimal alpha and lambda value (lambda.1se was selected for use).
2. The glmnet function from the 'glmnet' package was used to fit model using all available data and hyperparameters values optimized in the previous step.
3. Data were resampled 500 times, with all members of a single family allocated to the resampled dataset together to maintain family unit integrity. Hyperparameters were re-optimized again for each resample.
4. The glmnet function was applied with resampled data for training. Data of families that **were NOT** present in the i-th (resampled) dataset were used for estimation of out-of-sample AUC (validation). This was done for all resamples, totaling 500 iterations.
5. The average AUC and 2.5th and 97.5th percentiles were reported as out-of-sample AUC and its bounds of 95% confidence intervals.
6. Difference between out-of-sample AUCs of baseline vs. more complex model was calculated for each data resample, obtaining average difference in AUC (expressed as AUC_gain) and its 95% CI

References

Bates, D., Mächler, M., Bolker, B. M., & Walker, S. C. (2015). Fitting Linear Mixed-Effects Models Using lme4. *Journal of Statistical Software*, *67*(1), 1–48. https://doi.org/10.18637/JSS.V067.I01

Friedman, J., Hastie, T., & Tibshirani, R. (2010). Regularization Paths for Generalized Linear Models via Coordinate Descent. *Journal of Statistical Software*, *33*(1), 1–22. https://doi.org/10.18637/JSS.V033.I01

Gelman, A., & Su, Y.-S. (2024). Data Analysis Using Regression and Multilevel/Hierarchical Models [R package arm version 1.14-4]. *CRAN: Contributed Packages*. https://doi.org/10.32614/CRAN.PACKAGE.ARM

Kassambara, A. (2023). "ggplot2" Based Publication Ready Plots [R package ggpubr version 0.6.0]. *CRAN: Contributed Packages*. https://doi.org/10.32614/CRAN.PACKAGE.GGPUBR

Koller, M. (2016). robustlmm: An R Package for Robust Estimation of Linear Mixed-Effects Models. *Journal of Statistical Software*, *75*(1), 1–24. https://doi.org/10.18637/JSS.V075.I06

Rios-Leyvraz, M., & Yao, Q. (2023). The Volume of Breast Milk Intake in Infants and Young Children: A Systematic Review and Meta-Analysis. *Breastfeeding Medicine : The Official Journal of the Academy of Breastfeeding Medicine*, *18*(3), 188–197. https://doi.org/10.1089/BFM.2022.0281

Robin, X., Turck, N., Hainard, A., Tiberti, N., Lisacek, F., Sanchez, J. C., & Müller, M. (2011). pROC: An open-source package for R and S+ to analyze and compare ROC curves. *BMC Bioinformatics*, *12*(1), 1–8. https://doi.org/10.1186/1471-2105-12-77/TABLES/3

Selinger, E., Heniková, M., Světnička, M., Ouřadová, A., Cahová, M., Potočková, J., Dlouhý, P., Hrnčířová, D., el-Lababidi, E., & Gojda, J. (2024). Monitoring of emerging nutritional factors impacting health outcomes: KOMPAS prospective family cohort study. *MedRxiv*, 2024.03.03.24303671. https://doi.org/10.1101/2024.03.03.24303671

van Buuren, S., & Groothuis-Oudshoorn, K. (2011). mice: Multivariate Imputation by Chained Equations in R. *Journal of Statistical Software*, *45*(3), 1–67. https://doi.org/10.18637/JSS.V045.I03

Vignerová, J., & Lhotská, L. (2006). A fresh look at growth assessment of infants and young children in the Czech Republic in context of international developments. *Central European Journal of Public Health*, *14*(2), 97–100. https://doi.org/10.21101/cejph.a3371

Wood, S. N. (2011). Fast stable restricted maximum likelihood and marginal likelihood estimation of semiparametric generalized linear models. *Journal of the Royal Statistical Society: Series B (Statistical Methodology)*, *73*(1), 3–36. https://doi.org/10.1111/J.1467-9868.2010.00749.X
