# Supplementary material for: Dietary intake, nutritional status, and health outcomes among vegan, vegetarian, and omnivorous Czech families
Source: Commun Med (Lond). 2025 Nov 22;5:538. doi: 10.1038/s43856-025-01257-z (PMC12748659; doi:10.1038/s43856-025-01257-z)
Supplement: Supplementary file 3 — Description of Additional Supplementary files [file 43856_2025_1257_MOESM3_ESM.pdf]

Description of Additional Supplementary Files

File name: Supplementary Data 1

Description: Laboratory Analytic Methods
