# Supplementary material for: Dietary intake, nutritional status, and health outcomes among vegan, vegetarian, and omnivorous Czech families
Source: Commun Med (Lond). 2025 Nov 22;5:538. doi: 10.1038/s43856-025-01257-z (PMC12748659; doi:10.1038/s43856-025-01257-z)
Supplement: Supplementary file 4 — Supplementary Data 1 [file 43856_2025_1257_MOESM4_ESM.docx]

# Supplementary Data 1

Title: **Dietary intake, Nutritional status, and Health outcomes among Vegan, Vegetarian and Omnivorous Czech families**

Marina Heniková ^1,2, *^, Anna Ouřadová ^1 *^, Eliška Selinger ^1,3, 4^, Filip Tichanek ^4^, Petra Polakovičová ^4^, Dana Hrnčířová ^2^, Pavel Dlouhý ^2^, Martin Světnička ^5^, Eva El-Lababidi ^5^, Jana Potočková^1^, Tilman Kühn ^6^, Monika Cahová ^4†^, Jan Gojda ^1†^

*1/ Department of Internal Medicine, Kralovske Vinohrady University Hospital and Third Faculty of Medicine, Charles University, Prague, Czech Republic;*

*2/ Department of Hygiene, Third Faculty of Medicine, Charles University, Prague, Czech Republic;*

*3/ Department of Epidemiology, Third Faculty of Medicine, Charles University, Prague, Czech Republic;*

*4/ Centre for Public Health Promotion, National Health Institute, Prague, Czech Republic ;*

*5/ Institute for Clinical and Experimental Medicine, Prague, Czech Republic;*

*6/ Department of Pediatrics, Kralovske Vinohrady University Hospital and Third Faculty of Medicine, Charles University, Prague, Czech Republic;*

*7/ Department of Epidemiology, MedUni, Vienna, Austria.*

Correspondence:

Jan Gojda, MD, PhD., e-mail: [jan.gojda@lf3.cuni.cz](mailto:jan.gojda@lf3.cuni.cz), ORCID no. 0000-0002-7995-5947

*These authors have contributed equally and shall be considered as joint first authors

^†^ These authors jointly supervised this work

*Laboratory Analytic Methods.*

| Laboratory parameter | Analytic method | Analyzer | Reference |
| --- | --- | --- | --- |
| FPG (mmol/l) | absorption spectrophotometry | Siemens Atellica | 1D - 1M 1.67 - 4.44 mmol/l  1M - 1Y 3.3 - 5.5 mmol/l  1 - 15YRS 3.3 - 5.5 mmol/l  15 - 99YRS 3.6 - 5.59 mmol/l |
| TC (mmol/l) | absorption spectrophotometry | Siemens Atellica | 0D - 1M 1.3 - 4.3 mmol/l  1M - 1Y 2.6 - 4.2 mmol/l  1Y - 15YRS 2.6 - 4.8 mmol/l  15 - 99YRS 2.9 - 5 mmol/l |
| C-HDL (mmol/l) | absorption spectrophotometry | Siemens Atellica | 1D - 1M 0.73 - 1.17 mmol/l  1M - 1Y 0.94 - 1.26 mmol/l  1Y - 10YRS 1.11 - 1.83 mmol/l  10 - 15YRS 1.27 - 1.71 mmol/l  15 - 99YRS  F 1.2 - 2.7 mmol/l  M 1 - 2.1 mmol/l |
| C-LDL (mmol/l) | formula | calculated using the standard Friedewald equation | 0D - 5YRS 0.5 - 1.5 mmol/l  5 - 18YRS 1.6 - 3 mmol/l  18 - 99YRS 1.2 - 3 mmol/l |
| TG (mmol/l) | absorption spectrophotometry | Siemens Atellica | 0D - 1M 0.8 - 1.2 mmol/l  1M - 1Y 0.9 - 2.2 mmol/l  1Y - 15YRS 1.2 - 1.6 mmol/l  15 - 99YRS 0.45 - 1.7 mmol/l |
| Ca (mmol/l) | atomic absorption spectroscopy | Siemens Atellica | 0D - 1Y 2.2 - 2.84 mmol/l  1Y - 14YRS 2.25 - 2.69 mmol/l  14 - 19YRS  F 2.2 - 2.62 mmol/l  M 2.3 - 2.77 mmol/l  19 - 99YRS 2 - 2.75 mmol/l |
| P (mmol/l) | absorption spectrophotometry | Siemens Atellica | 0D - 1M 1.36 - 2.58 mmol/l  1M - 1Y 1.29 - 2.26 mmol/l  1Y - 15YRS 1.16 - 1.9 mmol/l  15 - 99YRS 0.65 - 1.61 mmol/l |
| Mg (mmol/l) | absorption spectrophotometry | Siemens Atellica | 1D - 1M 0.75 - 1.15 mmol/l  1M - 1Y 0.66 - 0.95 mmol/l  1Y - 15YRS 0.78 - 0.99 mmol/l  15 - 99YRS 0.7 - 1 mmol/l |
| Se (μmol/l) | Inductively coupled plasma mass spectrometry | Shimadzu ICPMS-2030 | 0D - 100YRS 0.75-1.86 μmol/l |
| Zn (μmol/l) | Inductively coupled plasma mass spectrometry | Shimadzu ICPMS-2030 | 0D - 4MS 9.9 - 21.4 μmol/l  4MS - 1Y 9.9 - 19.9 μmol/l  1Y - 5YRS 10.3-18.1 μmol/l  5 - 10YRS 11.8-16.4 μmol/l  10 - 15YRS 9.8-18 μmol/l  15 - 100YRS 9.8-18 μmol/l |
| Iron (µmol/l) | absorption spectrophotometry | Siemens Atellica | 0D - 1M 11 - 36 µmol/l  1M - 1Y 6 - 28 µmol/l  1Y - 15YRS 4 - 24 µmol/l  15 - 99YRS  F 6.6 - 28 µmol/l  M 7.2 - 29 µmol/l |
| TIBC (µmol/l) | formula | TIBC (µmol/l) = Transferrin (g/l) × 25.2 | 0D - 99YRS  F 24.2 - 70.1 µmol/l  M 22.3 - 61.7 µmol/l |
| Ferritin (µg/l) | chemiluminescent immunoassay | Siemens Atellica | 1Y - 15YRS 7 - 142 µg/l  15 - 99YRS  F 10 - 291 µg/l  M 22 - 322 µg/l |
| Transferrin (g/l) | turbidimetric immunoassay | Siemens Atellica | 0D - 1M 0.92 - 2.08 g/l  1M - 1Y 1.28 - 3.64 g/l  1Y - 15YRS 1.71 - 3.74 g/l  15 - 99YRS 2 - 3.6 g/l |
| Transferrin saturation (%) | formula | Transferin saturation (%) = (3.98 × Serum iron (µmol/l) ) / Transferrin (g/l) | 0D - 99YRS 16 - 45 % |
| sTfR Index | formula | Soluble transferrin receptor (sTfR)/log ferritin index | 0.76-1.76 |
| STFR (mg/l) | nephelometry | Siemens Atellica NEPH 630 | 15YRS - 99YRS 0.76 - 1.76 mg/l |
| Hb (g/l) | absorption spectrophotometry | Sysmex XN | 2WS - 4WS 125 - 205 g/l  4WS - 2MS 100 - 180 g/l  2 - 3MS 90 - 140 g/l  3 - 6MS 95 - 135 g/l  6MS - 2YRS 105 - 135 g/l  2 - 6YRS 115 - 135 g/l  6 - 12YRS 115 - 155 g/l  12 - 15YRS  F 120 - 160 g/l  M 130 - 160 g/l  15 - 99YRS  F 120 - 160 g/l  M 135 - 175 g/l |
| MCV (fl) | formula | Sysmex XN MCV = (hematocrit/ RBC count) | 0D - 3DS 95 - 121 fl  4DS - 2WS 88 - 126 fl  2 - 4WS 86 - 124 fl  4WS - 2MS 85 - 123 fl  2 - 3MS 77 - 115 fl  3 - 6MS 74 - 108 fl  6MS - 2YRS 70 - 86 fl  2 - 6YRS 75 - 87 fl  6 - 12YRS 77 - 95 fl  12 - 15YRS  F 78 - 102 fl  M 78 - 98 fl  15 - 99YRS+  F 82 - 98 fl  M 82 - 98 fl |
| PTH (pmol/l) | electrochemiluminescence immunoassay | PTH 1-84 - Roche Cobas e411 | 0D - 99YRS 1.6 - 6 pmol/l |
| Beta cross laps (µg/l) | electrochemiluminescence immunoassay | Roche Cobas e801 | < 5YRS 0.600 - 1.700 µg/l  5 - 9YRS 0.700 - 1.900 µg/l  F  9 - 14YRS 0.900 - 2.600 µg/l  14 - 16YRS 0.400 - 2.000 µg/l  16 - 17YRS 0.400 - 1.500 µg/l  17 - 20YRS 0.400 - 1.200 µg/l  20 - 23YRS 0.300 - 1.200 µg/l  23 - 26YRS 0.200 - 1.000 µg/l  26 - 30YRS 0.148 - 0.967 µg/l  30 - 40YRS 0.150 - 0.635 µg/l  40 - 50YRS 0.131 - 0.670 µg/l  50 - 60YRS 0.183 - 1.060 µg/l  60 - 70YRS 0.171 - 0.970 µg/l  70 - 150YRS 0.152 - 0.858 µg/l  M  9 - 11YRS 0.900 - 2.400 µg/l  11 - 13YRS 1.000 - 3.000 µg/l  13 - 15YRS 1.000 - 3.300 µg/l  15 - 16YRS 1.000 - 3.000 µg/l  16 - 17YRS 0.750 - 2.700 µg/l  17 - 19YRS 0.500 - 2.300 µg/l  19 - 30YRS 0.238 - 1.019 µg/l  30 - 40YRS 0.225 - 0.936 µg/l  40 - 50YRS 0.182 - 0.801 µg/l  50 - 60YRS 0.161 - 0.737 µg/l  60 - 70YRS 0.132 - 0.752 µg/l  70 - 150YRS 0.118 - 0.776 µg/l |
| P1NP (µg/l) | electrochemiluminescence immunoassay | Roche Cobas e801 | F  < 1YRS 600 - 3000 µg/l  1 - 2YRS 600 - 2000 µg/l  2 - 3YRS 300 - 1300 µg/l  3 - 9YRS 300 - 950 µg/l  9 - 14YRS 300 - 1200 µg/l  14 - 15YRS 150 - 800 µg/l  15 - 17YRS 30 - 500 µg/l  17 - 18YRS 30 - 300 µg/l  18 - 23YRS 30 - 150 µg/l  23 - 26YRS 20 - 100 µg/l  26 - 150YRS 16.27 - 73.87 µg/l  M  < 1YRS 600 - 3000 µg/l  1 - 2YRS 600 - 2000 µg/l  2 - 3YRS 300 - 1300 µg/l  3 - 11YRS 300 - 950 µg/l  11 - 16YRS 300 - 1400 µg/l  16 - 19YRS 150 - 850 µg/l  19 - 150YRS 16.00 - 62.60 µg/l |
| Urea (mmol/l) | absorption spectrophotometry | Siemens Atellica | 0D - 1M 0.7 - 5 mmol/l  1M - 1Y 0.4 - 5.4 mmol/l  1Y - 15YRS 1.8 - 6.7 mmol/l  15YRS - 99YRS  F 2 - 6.7 mmol/l  M 2.8 - 8 mmol/l |
| Creatinine (µmol/l) | absorption spectrophotometry | Siemens Atellica | 1D - 1M 12 - 48 µmol/l  1M - 1Y 21 - 55 µmol/l  1Y - 15YRS 27 - 88 µmol/l  15YRS - 99YRS+  F 46 - 90 µmol/l  M 63 - 104 µmol/l |
| UA (µmol/l) | absorption spectrophotometry | Siemens Atellica | 1D - 1M 143 - 340 µmol/l  1M – 1Y 120 - 340 µmol/l  1Y - 15YRS 140 - 340 µmol/l  15YRS - 99YRS+  F 140 - 340 µmol/l  M 220 - 420 µmol/l |
| Holotranscobalamine (pmol/l) | chemiluminescence immunoassay | Siemens Atellica | 0D - 99YRS 27.24 - 169.62 pmol/l |
| Homocysteine (µmol/l) | chemiluminescent immunoassay | Siemens Atellica | 0D - 99YRS 4 - 15.2 µmol/l |
| MMA (nmol/l) | liquid chromatography-mass spectrometry | Agilent 6460 TripleQuad LC/MS System | 0D - 100YRS 80.0-560.0 |
| 25-hydroxyvitamin D (nmol/l) | chemiluminescent immunoassay | DiaSorin Inc. USA | 0D - 99YRS 75 - 250 nmol/l |
| Folate (µg/l) | chemiluminescent immunoassay | Siemens Atellica | 0D - 99YRS 5.38 - 40 µg/l |
| IGF-1 (µg/l) | electrochemiluminescence immunoassay | DiaSorin Inc. Liaison XL | F  2 - 5YRS 33.5 - 171.8 µg/l  6 - 8YRS 79.8 - 244 µg/l  9 - 11YRS 87.4 - 399.3 µg/l  12 - 15YRS 188.4 - 510 µg/l  16 - 20YRS 267.5 - 470.8 µg/l  20 - 24YRS 149.1 - 332.3 µg/l  25 - 39YRS 107.8 - 246.5 µg/l  40 - 54YRS 92.7 - 244.6 µg/l  54 - 100YRS 54 - 204.4 µg/l  M  2 - 5YRS 27.4 - 113.5 µg/l  6 - 8YRS 54.9 - 206.4 µg/l  9 - 11YRS 85.2 - 248.8 µg/l  12 - 15YRS 115.4 - 498.2 µg/l  16 - 20YRS 247.3 - 481.7 µg/l  20 - 24YRS 187.9 - 400 µg/l  25 - 39YRS 96.4 - 227.8 µg/l  40 - 54YRS 88.3 - 209.9 µg/l  54 - 100YRS 54.6 - 185.7 µg/l |
| TSH (mIU/l) | chemiluminescent immunoassay | Siemens Atellica | 1M – 2YRS 0.87 - 6.15 mIU/l  2 – 12YRS 0.67 - 4.16 mIU/l  12 – 20YRS 0.48 - 4.17 mIU/l  20 – 99+YRS 0.55 - 4.78 mIU/l |
| uCreatinine (mmol/l) | absorption spectrophotometry | Siemens Atellica | Not specified |
| UIC (µg/l) | inductively coupled plasma mass spectrometry | Shimadzu ICPMS-2030 | Not specified |
| uICR (µg/g) | formula |  | Not specified |
| uCalcium (mmol/l) | absorption spectrophotometry | Siemens Atellica | Not specified |
| uCCR | formula |  | 0D - 99YRS 0.25 - 0.55 (mg/mg) |
| uPhosphorus (mmol/l) | absorption spectrophotometry | Siemens Atellica | Not specified |
| uPCR | formula |  | Notspecified |

Reference per the laboratory manuals of the Laboratory diagnostics department of University Hospital Kralovske Vinohrady (ČSN EN ISO 15189; available online <https://fnkv.slp.blue/verejna-prirucka/stranka/laboratorni-prirucka-centralnich-laboratori-fnkv>) and Laboratory Spadia (ČSN EN ISO 15189:2013; available online https://virtuallab.spadia.cz/Verejne/LaboratorniPrirucka) for PTH, CTx, P1NP, UIC, MMA, IGF-1. FPG, Fasting plasma glucose; TC, Total cholesterol; C-HDL, HDL cholesterol; C-LDL, LDL cholesterol; TG, Triglycerides; Ca, Calcium; P, Phosphate; Mg, Magnesium; Se, Selenium; Zn, Zinc; TIBC, Total iron binding capacity; sTfR Index, Soluble transferrin receptor/log Ferritin Index; STFR, Soluble transferrin receptor; Hb, Hemoglobine; MCV, Mean corpuscular volume; PTH, Parathormone; CTx, Beta cross laps; P1NP, Procollagen type I aminoterminal propeptide; UIC, Urine iodine concentration; UA, Uric acid; Active B12, Holotranscobalamine; MMA, Methylmalonic acid; IGF-1, Insulin-like growth factor 1; uCr, Urinary Creatinine; uCa, Urinary Calcium; uCCR, Urinary calcium-creatinine ratio; uICR, Urinary iodine-creatinine ratio; uP, Urinary phosphate; uPCR, Urinary phosphate-creatinine ratio.
